# Triggering Bimodal Radial Stem Growth in *Pinus sylvestris* at a Drought-Prone Site by Manipulating Stem Carbon Availability

**DOI:** 10.3389/fpls.2021.674438

**Published:** 2021-05-28

**Authors:** Walter Oberhuber, Anton Landlinger-Weilbold, Dennis Marko Schröter

**Affiliations:** Department of Botany, Leopold-Franzens-University of Innsbruck, Innsbruck, Austria

**Keywords:** bimodal growth, cambial activity, carbon availability, drought, girdling, radial growth, Scots pine, wood anatomy

## Abstract

A bimodal radial growth (RG) pattern, i.e., growth peaks in spring and autumn, was repeatedly found in trees in the Mediterranean regions, where summer drought causes reduction or cessation of cambial activity. In a dry inner Alpine valley of the Eastern Alps (Tyrol, Austria, 750 m asl), *Pinus sylvestris* shows unimodal RG with onset and cessation of cambial activity in early April and late June, respectively. A resumption of cambial activity after intense summer rainfall was not observed in this region. In a field experiment, we tested the hypothesis that early cessation of cambial activity at this drought-prone site is an adaptation to limited water availability leading to an early and irreversible switch of carbon (C) allocation to belowground. To accomplish this, the C status of young *P. sylvestris* trees was manipulated by physical blockage of phloem transport (girdling) 6 weeks after cessation of cambial cell division. Influence of manipulated C availability on RG was recorded by stem dendrometers, which were mounted above the girdling zone. In response to blockage of phloem flow, resumption of cambial activity was detected above girdling after about 2 weeks. Although the experimentally induced second growth surge lasted for the same period as in spring (*c*. 2 months), the increment was more than twice as large due to doubling of daily maximum RG rate. After girdling, wood anatomical traits above girdling no longer showed any significant differences between earlywood and latewood tracheids indicating pronounced effects of C availability on cell differentiation. Below girdling, no reactivation of cambial activity occurred, but cell wall thickness of last formed latewood cell was reduced due to lack of C supply after girdling. Intense RG resumption after girdling indicates that cessation of cambial activity can be reversed by manipulating C status of the stem. Hence, our girdling study yielded strong support for the hypothesis that belowground organs exert high C sink strengths on the drought-prone study site. Furthermore, this work highlights the need of in-depth experimental studies in order to understand the interactions between endogenous and exogenous factors on cambial activity and xylem cell differentiation more clearly.

## Introduction

Drought stress is a common trigger of growth reduction or premature cessation of cambial activity and cell differentiation in trees (e.g., van der Werf et al., 2007; Camarero et al., 2010; De Luis et al., 2011; Balducci et al., 2013), because cambial activity and cell differentiation are highly responsive to water availability (Sterck et al., 2008; Muller et al., 2011; Deslauriers et al., 2016). In several Mediterranean tree species (*Pinus* spp., *Juniperus thurifera*, *Quercus ilex*, and *Arbutus unedo*), cambial activity resumes, i.e., is reactivated in autumn if soil water availability increases again after prolonged summer drought (De Luis et al., 2007; Battipaglia et al., 2010; Camarero et al., 2010; Gutiérrez et al., 2011; Pacheco et al., 2016; Campelo et al., 2018). This bimodal pattern of cambial activity leads to the formation of intra-annual density fluctuations (IADFs; e.g., Campelo et al., 2007; Novak et al., 2013; Battipaglia et al., 2016; De Micco et al., 2016; Pacheco et al., 2018). The formation of earlywood-like tracheids with wide lumen and thin cell walls in latewood (also called L-IADF; Campelo et al., 2013) reflects the ability of some tree species to respond to favorable environmental conditions at the end of the growing season. However, IADF formation is not obligatorily linked to bimodal growth, because short-term fluctuations in cambial activity and cell enlargement during the growing period can also induce formation of IADFs in trees of temperate climate zone (Wimmer et al., 2000; Rigling et al., 2002; Vieira et al., 2009; Rozas et al., 2011).

Several dendroecological studies conducted at a drought-prone inner Alpine site revealed that limited soil water availability in spring and early summer constrains radial stem growth (RG) in coniferous species (e.g., Rigling et al., 2002; Zweifel et al., 2006; Schuster and Oberhuber, 2013). Analyses of intra-annual dynamics of RG and wood formation by dendrometers and microcoring, respectively, revealed that the maximum RG rate of co-occurring conifers peaked early in the growing season in late May through early June (Gruber et al., 2010; Oberhuber et al., 2014), although higher precipitation in summer would provide more favorable environmental conditions for tree growth. Gruber et al. (2010) suggested that the early decrease in RG might be due to an early shift of carbon (C) allocation to belowground organs as an adaptation to ameliorate drought stress. This view is corroborated by several authors who reported that (*i*) plant growth is limited by competition between sinks rather than directly by C resources (e.g., Körner, 2003; Simard et al., 2013; Guillemot et al., 2017) and (*ii*) that the mycorrhiza-associated root system is a strong sink for C in plants experiencing water shortage during the growing period (Shipley and Meziane, 2002; Brunner et al., 2015; Hagedorn et al., 2016; Rainer-Lethaus and Oberhuber, 2018; Hartmann et al., 2020).

Wood formation is a highly C-demanding process (Koch, 2004; Simard et al., 2013; Cuny et al., 2015; Deslauriers et al., 2016). Therefore, physical blockage of phloem transport (girdling) allows evaluation of the influence of changes in tree C status on RG (Maier et al., 2010; Maunoury-Danger et al., 2010; De Schepper and Steppe, 2011; Rademacher et al., 2019). Previously, we found that in potted Norway spruce (*Picea abies*) saplings exposed to drought, cambial reactivation and intense RG occurred above girdling (Oberhuber et al., 2017). However, a field study determining RG response and effects on xylem cell differentiation of drought-stressed *Pinus sylvestris* to phloem blockage after cessation of RG is still lacking. Due to higher sensitivity of growth processes compared to photosynthesis to drought (Muller et al., 2011), carbohydrates accumulate during periods of water shortage and C accumulation during drought could modulate wood formation and growth dynamics during resumption of cambial activity. By applying automatic dendrometers, intra-annual dynamics of RG can be followed at high-resolution pre- and post-girdling, i.e., during the regular and induced growing period, respectively.

Aims of this study therefore were (*i*) to assess whether phloem blockage after cessation of RG and shoot growth induces reactivation of cambial activity in young *P. sylvestris* trees at a xeric site in the field, (*ii*) to analyze differences in dynamics (growth rate, duration, and intensity) of RG before and after phloem blockage, and (*iii*) to compare wood anatomical traits [cell lumen diameter (CLD), cell area (CA), and cell wall thickness] between regular spring growth and the induced growth phase after girdling. We hypothesized that cambial reactivation after girdling occurs and due to increase in C availability above girdling, induced RG in summer shows different kinetics, i.e., increase in duration, growth rate, and total increment, compared with regular spring RG. Furthermore, we expected a crucial role of higher C availability after phloem blockage on wood anatomical traits, especially an increase in cell wall thickness.

## Materials and Methods

### Study Site

The field study was conducted at a xeric site at 750 m asl in a dry inner Alpine valley of the Eastern Alps in Austria (47° 13′ 53″ N, 10° 50′ 51″ E). Based on >100 years of climate records at Ötz (812 m asl, 5 km from the study area) mean annual air temperature and total precipitation amount to 7.3°C and 724 mm, respectively (long-term means during 1911–2017). According to the FAO classification system (FAO, 2006) soils of the protorendzina type, i.e., rendzic and lithic leptosols, are primarily developed. As a result of low soil depth and the coarse-textured structure, soils have a low water holding capacity. In this drought-prone environment, Scots pine (*Pinus sylvestris* L.) dominates and forms poorly growing open stands (Erico-Pinetum typicum, Ellenberg and Leuschner, 2010). We selected a south-west facing steep slope (*c*. 30°), where *P. sylvestris* rejuvenates naturally under open canopy. In order to minimize the impact on the protected forest area, we used young trees for the girdling experiment instead of mature trees. Stem height and diameter of selected trees (*n* = 7) amounted to 1.5 m and 2.9 cm, respectively. Age of trees at 30 cm stem height was 35 ± 5 years (Table 1).

**TABLE 1:** Site description and characteristics of selected *Pinus sylvestris* trees (*n* = 7).

**Site**	**Aspect**	**Slope (°)**	**CC (%)**	**Soil type**	**Humus type**	**Soil depth (cm)**	**Tree age^1^ (year) mean ± SD**	**Stem height^2^ (m)**	**SDM^2,3^ (cm) mean ± SD**
Xeric	SW	30	33	Syrosem	Xeromoder	<10	35 ± 5	1.5 ± 0.3	2.9 ± 0.6

*Mean values ± standard deviation (SD) are presented (CC, canopy coverage and SDM, stem diameter).*

*^1^Cambial age at height of dendrometers.*

*^2^Measured at start of dendrometer records in March 2019.*

*^3^Measured at height of dendrometers.*

### Phloem Blockage

Girdling was applied to block phloem C transport from aboveground to belowground sinks. Trees (*n* = 3) were girdled after cessation of shoot and RG in mid-July 2019 (day of the year (doy 199) at a stem height of 25 cm above the soil surface by carefully detaching a 2–3-cm-wide band of bark including periderm, living phloem, and cambium. Exposed wood was treated with a fat-containing cream to prevent dehydration. At the time of girdling, cambial cell division of trees has already stopped for about 6 weeks (cf. Figure 2).

### Dendrometer Records

Temperature compensated electronic diameter dendrometers with resolutions of <3 μm (DD-S, Ecomatik, Munich, Germany) were installed on trees (controls: *n* = 4; girdled trees: *n* = 3) at 5–7 cm above girdling to record intra-annual dynamics of RG. The temperature coefficient of the sensor was <0.2 μm/K (unverified information of the producer). The dead outermost loose layer of the bark (periderm) was slightly removed to allow proper mounting of the dendrometer and to ensure close contact with the stem. Data were recorded every 30 min with analog data loggers (HOBO UX120-006M, ONSET, Bourne, MA, United States).

Daily stem diameter variations (SDVs) were calculated by averaging all daily measurements (48 values per day), i.e., one value per day was extracted from the time series. The *daily mean approach* yields time series of daily SDVs, which consist of both water- and growth-induced diameter changes (Deslauriers et al., 2007). It has to be considered that irreversible growth-related diameter increments recorded by dendrometers include formation of xylem, phloem, and outer bark (Plomion et al., 2001). Time series of daily SDVs were set to zero on March 1, and we modeled short-term variations in intra-annual RG with the Gompertz function, which is commonly used to describe RG dynamics in trees (Zeide, 1993; Rossi et al., 2003; De Micco et al., 2019), by applying the Origin software package (OriginLab Corporation, Northampton, MA, United States).

### Microclimate Records

**FIGURE 1:**
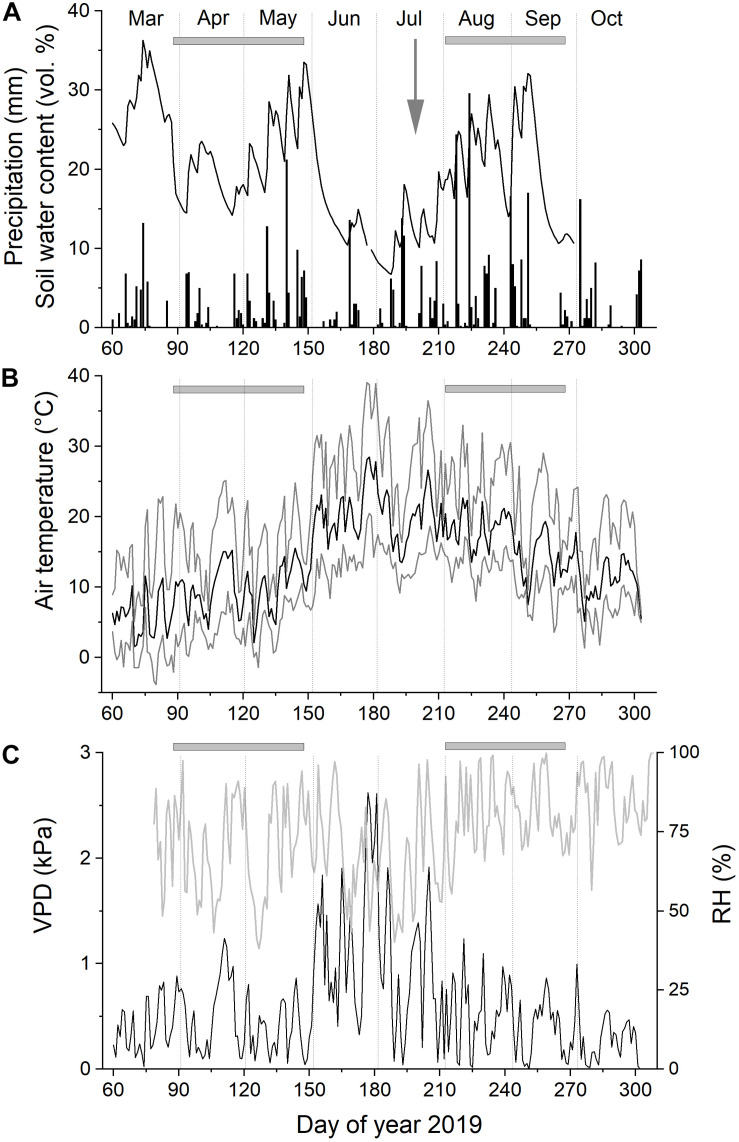
Climate variables and soil water content recorded at the study plot from March through October 2019. Daily precipitation and soil water content **(A)**, mean daily air temperature **(B)**, and relative air humidity (RH) and vapor pressure deficit of the air (VPD; **C**). Gray horizontal bars indicate regular (pre-girdling) and induced (post-girdling) growing periods in April–May and August–September, respectively (cf. Figure 2). Gray arrow in **(A)** indicates time of girdling at doy 199.

**FIGURE 2:**
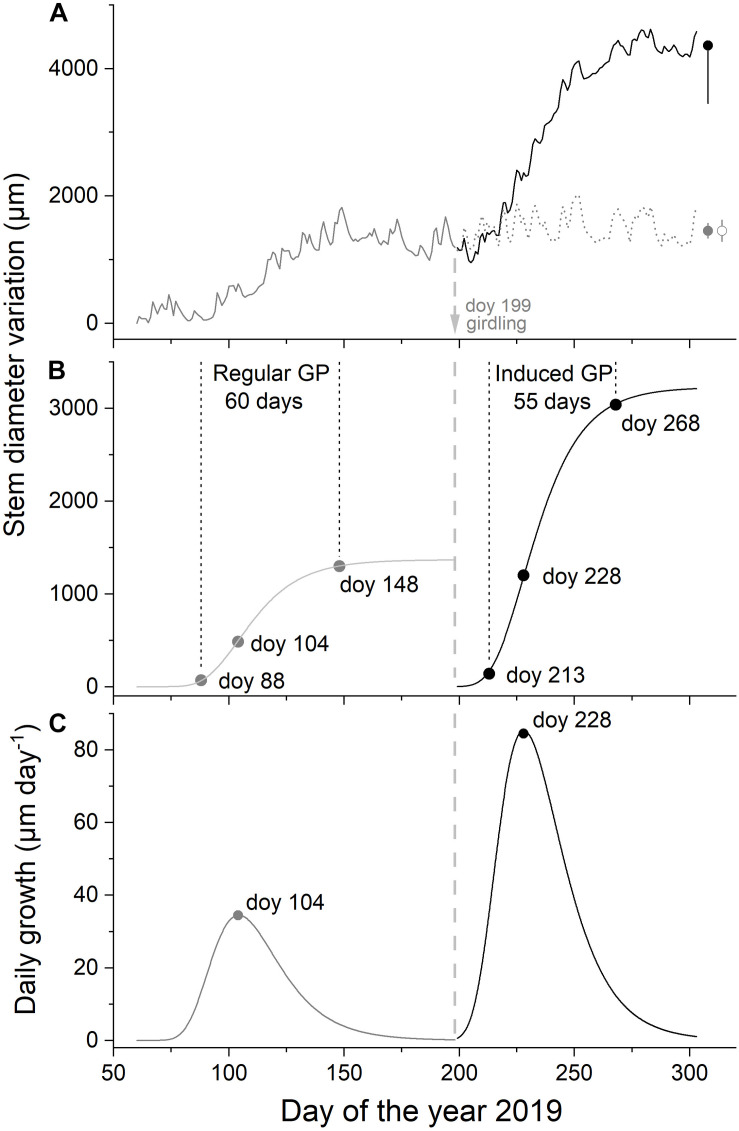
Time series of mean daily dendrometer records. Regular growth (pre-girdling, *n* = 7) and induced growth (post-girdling, *n* = 3) are given in gray and black lines, respectively. Dendrometer record of the non-girdled subset of trees (*n* = 4) is shown in dashed gray line **(A)**, modeled intra-annual radial growth by applying the Gompertz function in (**B**; for parameters, see Table 2), and daily growth pre-girdling and post-girdling calculated from Gompertz function in (**C**; GP, growth period). Arrow indicates time of girdling. Bars in **(A)** depict standard errors of mean daily dendrometer records during the last 25 days.

At the study site, environmental conditions [air temperature, relative air humidity (RH), and daily precipitation] were continuously monitored during the study period (March through October 2019) at 2 m aboveground (ONSET, Pocasset, MA, United States). In addition, three soil moisture sensors (ThetaProbes Type ML2x, Delta-T, Cambridge, England) were installed at 5–10 cm soil depth to record changes in volumetric soil water content (SWC). In data loggers, measuring intervals for all sensors were programmed to 30 min. All measurements per day (i.e., 48 values) were used to calculate mean daily air temperature and relative air humidity (RH). The equation presented in Prenger and Ling (2000) was used to compute vapor pressure deficit of the air (VPD). Climate records and SWC during the study period are depicted in Figure 1. Student’s *t* test was applied to detect significant differences among climate and environmental variables during the regular (i.e., pre-girdling) and induced (i.e., post-girdling) growing period (cf. Figure 2).

### Wood Anatomy

Changes in wood anatomy (radial CLD; CA; and radial cell wall thickness, CWT) in response to C manipulation were analyzed at the end of the growing season. Stem cross-sections were collected from all trees (controls and girdled trees) at the position of dendrometers. In girdled trees additional cross-sections were sampled 5 cm above and below the girdling zone. The chosen distance to the girdling zone ensured that influence of wound responses on wood formation was avoided. CWT, CA, and CLD were measured in earlywood and latewood on stem transverse sections of ∼20-μm thickness, which were cut using a sliding microtome. To distinguish between earlywood and latewood tracheids, the Mork’s index, i.e., the ratio between the double CWT and CLD (both measured in radial direction), was computed (Denne, 1988). In non-girdled controls (*n* = 4 trees) and in girdled trees (*n* = 3 trees), anatomical features of tracheids were determined in earlywood and latewood pre- and post-girdling. Additionally, continuous records of wood anatomical parameters were determined above and below girdling and in non-girdled controls along five radial cell rows per tree, and mean values were calculated. In these time series time of girdling was deduced from intra-annual wood formation dynamics determined in *P. sylvestris* within the study area (Gruber et al., 2010) and abrupt alterations in anatomical traits. Because separation of individual cell walls could not be unequivocally detected in all samples, CWT of adjacent cells was recorded (double radial CWT), and this value was then halved yielding radial CWT. Cell anatomical parameters were measured by applying the image analysis software PROGRES Gryphax (Version 2.0.0.68, Jenoptik Optical Systems GmbH, Jena, Germany) and were recorded throughout five earlywood and latewood cells along five cell rows (i.e., *n* = 25 cells per sample), and mean values and standard deviations were calculated. The proportion of cell wall material was calculated as the ratio between CLD and CWT. A decrease/increase in CLD:CWT indicates an increase/decrease in wood density. Student’s dependent sample *t* test was used to determine significant differences among cell anatomical traits pre- and post-girdling and non-girdled controls.

## Results

At the start of the growing season in late March/early April, mean daily air temperature and SWC reached 10°C and >20 vol%, respectively (Figures 1A,B). After frequent rainfalls in May, SWC rose to >30 vol%. Only sporadic rainfall during June and July caused SWC to drop to c. 10 vol%. Mean daily air temperature and VPD reached highest values during late June (28°C and 2.6 kPa, respectively; Figures 1B,C). Starting with rainfall events in mid-July, SWC reached again >30 vol% in early September. The observed abrupt drops in SWC following precipitation events are caused by low water holding capacity of the shallow, stony soils prevailing at the study plot (Table 1). Mean SWC during the regular and induced RG period (see Figure 2) amounted to about 21% (Table 2). Mean air temperature during these periods was 9.8 ± 3.3°C (regular RG period) and 16.5 ± 3.5°C (induced RG period). Precipitation was 31% higher during the induced RG period compared with the regular RG period (Table 2). Other environmental variables recorded at the study plot (SWC, VPD, and RH) were not significantly different between RG periods.

**TABLE 2:** Climate variables and soil water content during the regular (i.e., pre-girdling) and induced (i.e., post-girdling) growing period (cf. Figure 2).

**Growing period**	**Precipitation (mm)**	**SWC (vol.%)**	**Air temp (°C)**	**VPD (kPa)**	**RH (%)**
Regular	124	21.1 ± 4.7^a^	9.8 ± 3.3^a^	0.457 ± 0.31^a^	71.6 ± 15.8^a^
Induced	162	21.1 ± 6.1^a^	16.5 ± 3.5^b^	0.460 ± 0.31^a^	82.2 ± 10.1^a^

*Except for precipitation (total sum), mean values ± standard deviations are shown (SWC, soil water content; Air temp, mean air temperature; VPD, vapor pressure deficit; and RH, relative air humidity). Different letters indicate significant differences (Student’s *t* test; *P* < 0.001) between growing periods.*

Dendrometer traces and Gompertz growth models pre- and post-girdling are depicted in Figure 2. Regular growth period started end of March (doy 88), reached a maximum in mid-April (doy 104), and ceased end of May (doy 148). Girdling in mid-July (doy 199) of a subset of trees induced a second growth surge after about 2 weeks (doy 213), which exceeded regular RG in spring more than twofold (Figure 2 and Table 3). During regular and induced RG, the inflection point of modeled growth was reached about 2 weeks after growth onset (Table 3). While growth duration amounted to about 2 months during both growing periods, growth intensity (i.e., increment) and growth rate (i.e., slope of modeled growth at the inflection point) were quite different during regular and induced RG period. These parameters more than doubled post-girdling (Table 3).

**TABLE 3:** Parameters of the Gompertz function for radial growth dynamics of trees during the regular (i.e., pre-girdling) and induced (i.e., post-girdling) growing period (cf. Figure 2) and *R*^2^ of the model (*A*, upper asymptote; *I*_*p*_, inflection point; κ, rate of change parameter; and Growth_max_, maximum daily growth at inflection point).

**Growing period**	***A* (mm)**	***I*_p_ (doy)**	**Growth_max_ (μm/day)**	**κ**	***R*^2^**
Regular	1.37 ± 0.03	104 ± 1.1	32.9	0.069 ± 0.007	0.886
Induced	3.23 ± 0.03	228 ± 0.4	83.6	0.072 ± 0.003	0.987

*Mean values ± standard deviations are shown.*

**FIGURE 3:**
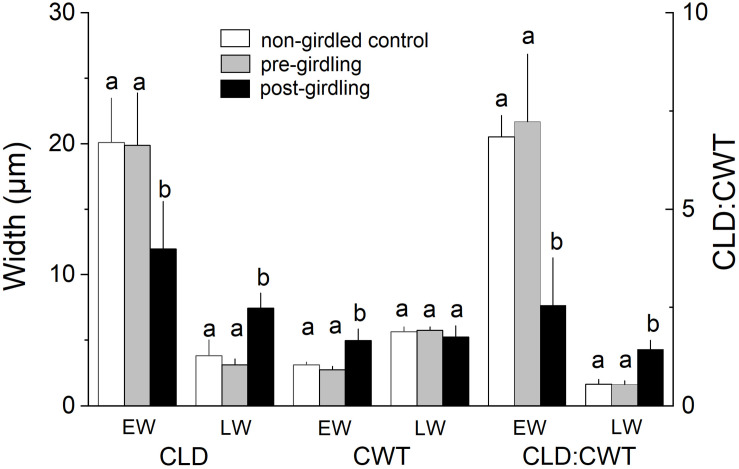
Radial cell lumen diameter (CLD), cell wall thickness (CWT), and ratio of CLD:CWT of earlywood (EW) and latewood (LW) tracheids in non-girdled controls, and above the girdling zone during the regular (i.e., pre-girdling) and induced growing period (i.e., post-girdling). Student’s *t* test was applied to determine statistically significant differences of anatomical parameters between non-girdled controls, and pre- and post-girdling. Different letters indicate significant difference at *P* < 0.05.

**FIGURE 4:**
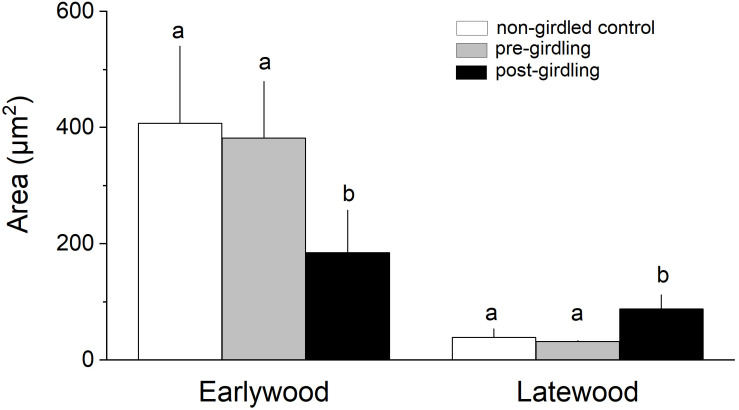
Cell area of earlywood (EW) and latewood (LW) tracheids in non-girdled controls, and above the girdling zone during the regular (i.e., pre-girdling) and induced growing period (i.e., post-girdling). Student’s *t* test was applied to determine statistically significant differences of anatomical parameters between non-girdled controls and pre- and post-girdling. Different letters indicate significant difference at *P* < 0.05.

**FIGURE 5:**
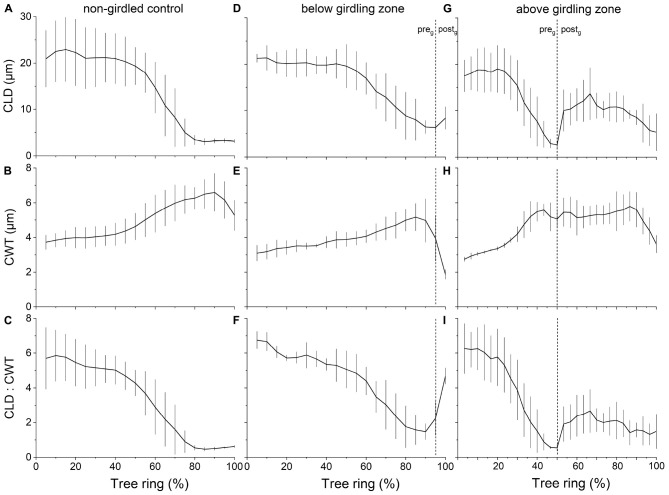
Change in wood anatomical parameters throughout growing period(s) in **(A–C)** non-girdled controls (*n* = 4) and **(D–F)** below and **(G–I)** above girdling (*n* = 3). In **(D–I)**, the regular (i.e., pre-girdling = pre_g_) and induced growing period (i.e., post-girdling = post_g_) are separated by dotted lines. Data were normalized to overall cell count of the individual tree in 2019. Mean values ± standard deviations are shown.

Measurement of wood anatomical parameters above the girdling zone revealed that after girdling CLD and CA significantly decreased in earlywood and increased in latewood, while CWT of tracheids developed after girdling significantly increased in earlywood (Figures 3, 4). After girdling, the ratio CLD:CWT decreased in earlywood (*P* = 0.019) and increased in latewood (*P* = 0.009). Wood anatomical parameters (CLD, CA, CWT, and CLD:CWT) were all significantly different between earlywood and latewood tracheids pre-girdling (*P* < 0.01), but not significantly different post-girdling (*P* > 0.05). Continuous measurements of cell anatomy revealed that below the girdling zone cambial activity did not resume after girdling (Figures 5A–F) and last latewood tracheid showed striking decrease in CWT (-52%) and slight increase in CLD leading to decrease in wood density (Figure 5F). Above the girdling zone CLD increased sharply after girdling (Figures 5G–I) and remained at about 50% of regular earlywood-CLD except for the last formed cells, which show decreasing CLD (Figure 5G). CWT stayed constant after girdling, i.e., it remained at the level of latewood-CWT again with the exception of last formed tracheids showing a decrease in CWT (Figure 5H).

## Discussion

Although frequently applied, girdling, i.e., removing surface tissues including outer bark, living phloem, and cambium around the stem, is a rather crude method to manipulate phloem transport compared with less-destructive methods like phloem compression or phloem chilling (Rademacher et al., 2019). Tangential rows of traumatic resin ducts and callus tissue are generally formed in conifers as a response to tissue damage (Schweingruber, 2007). Wood anatomical analyses of stem cross sections taken at the position of dendrometers, however, did not reveal presence of overgrowing callus tissue or abnormal formation of tangential rows of resin ducts after girdling, because we avoided possible wound reactions by analyzing growth response and wood anatomy 5–7 cm above the girdling zone. It has also been found that the less-destructive phloem chilling method leads to similar growth responses as girdling (De Schepper and Steppe, 2011), indicating that the applied method is a useful tool to infer growth response and changes in xylem cell differentiation provided the zone of direct wound response is avoided.

### Unimodal Pattern of Stem Radial Growth of *Pinus sylvestris* in Dry Inner Alpine Valleys

In contrast to tree species from the Mediterranean regions (e.g., Camarero et al., 2010; Battipaglia et al., 2016; Pacheco et al., 2018), which use adequate water availability in spring and autumn for tree growth, but show strongly reduced or no growth during the dry and hot summer period, a comparable bimodal growth pattern does not occur in *P. sylvestris* in dry inner Alpine environments. Several authors (Gruber et al., 2010; Oberhuber et al., 2014; Swidrak et al., 2014) reported a unimodal growth pattern characterized by early peak of RG in mid-May to early June in coniferous species (*P. sylvestris*, *P. abies*, and *Larix decidua*) within the study area, although extended dry periods frequently occur in spring and higher precipitation during summer would provide more favorable environmental conditions for tree growth. It was suggested that extreme environmental conditions, i.e., drought stress and nutrient deficiency of the predominantly dolomite bedrock cause an early shift in C allocation from aboveground stem growth to belowground sinks to ensure adequate resource acquisition (Oberhuber and Gruber, 2010; Swidrak et al., 2013). Dendrometer records of root RG of mature *P. sylvestris* trees having a diameter of approximately 10 mm also revealed unimodal growth lasting from June through early August (Supplementary Figure 1).

### Cambial Reactivation Induced by Girdling

Based on a previous experimental set-up using potted *P. abies* saplings (Oberhuber et al., 2017), we expected that girdling also triggers cambial reactivation of *P. sylvestris* under field conditions. Results of this study confirmed our hypothesis and revealed reactivation of cambial activity by phloem blockage 2 weeks after girdling at a drought-prone site. Girdling occurred in mid-July (doy 199), i.e., 6 weeks after cessation of the regular RG period excluding wall-thickening of latewood tracheids, which at xeric sites lasts until September (Gruber et al., 2010). Phloem blockage triggered cambial reactivation causing a bimodal RG pattern, i.e., a spring peak (doy 104) was followed by a secondary peak in summer (doy 228). While in Mediterranean areas bimodal growth is initiated by increase in water availability in autumn after intense summer drought, we could show that reactivation of cambial activity can be induced by an endogenous trigger, i.e., an increase in tree C status. In accordance with other studies (e.g., Daudet et al., 2005; De Schepper and Steppe, 2011; Oberhuber et al., 2017) results strongly suggest that interruption of C transport in the phloem to belowground sinks increased stem C availability above girdling inducing reactivation of cambial activity. This reasoning is corroborated by significant decrease in fine root mass of potted *P. abies* saplings in response to blockage of phloem C transport (Rainer-Lethaus and Oberhuber, 2018). Below girdling phloem blockage caused cessation of cell differentiation (evident in the form of reduced CWT of last latewood tracheids) and did not lead to a resumption of cambial activity. Hence, C availability in the stem, i.e., an endogenous factor is important for RG to occur under extreme environmental conditions. Based on findings of Smith and Stitt (2007) and Lastdrager et al. (2014), we suggest that sugar signaling—induced by interruption of phloem C transport—is involved in reactivation of cambial activity and triggering of bimodal RG in *P. sylvestris* trees.

Plant hormones direct growth and development and also responses to environmental stimuli (Davies, 2010). Accordingly, tree-ring formation, i.e., cambial activity and xylem cell differentiation, is induced and controlled by hormones (for a review see Buttò et al., 2020). Specifically, cambial activity is known to be highly responsive to the growth-promoting hormone auxin [indole-3-acetic acid (IAA); Uggla et al., 1998; Dünser and Kleine-Vehn, 2015; Bhalerao and Fischer, 2016] and Uggla et al. (1996) found a steep radial concentration gradient of IAA in Scots pine peaking in the cambium meristem. IAA is transported in the mature phloem in a basipetal flux from source tissues (leaf primordia and young leaves) to proximal regions (Muday and DeLong, 2001; Hacke et al., 2017). Several findings argue against the influence of accumulation of IAA above girdling on cambial reactivation: (*i*) heating of the stem can trigger cambial activity during dormancy (Oribe et al., 2003; Gričar et al., 2006; Begum et al., 2010), (*ii*) high levels of IAA are found in cambial tissues in the dormant period (Sundberg et al., 1990; Eklund et al., 1998; Funada et al., 2002), (*iii*) reduced responsiveness of cambial tissues to IAA during activity-dormancy transition (Baba et al., 2011), and (*iv*) sugar signaling can stimulate cambial cell division (e.g., Uggla et al., 2001; Lastdrager et al., 2014). The lag of about 2 weeks between girdling and onset of the induced RG period above girdling might be due to an extensive drought period at the time of girdling or as Oribe et al. (2003) suggested, that newly formed phloem cells are necessary for xylem formation to resume. The latter assumption is supported by results of Swidrak et al. (2014), who reported that phloem formation in *P. sylvestris* precedes xylem formation within the study area by about 3 weeks. In general, girdling induced cambial reactivation after regular spring growth was made possible because at the time of girdling the cambial meristem was not yet in endodormancy state (Lang et al., 1987; Chang et al., 2021), which can only be released by external triggers, i.e., chilling, photoperiod, and temperature (e.g., Badeck et al., 2004; Pallardy, 2008; Swidrak et al., 2011; Basler and Körner, 2014; Begum et al., 2018).

### Comparison of Regular vs. Induced Radial Stem Growth

The increase of C availability above girdling strongly affected RG rate and intensity (increase by 154 and 136%, respectively, compared with regular RG), while RG duration remained quite constant amounting to about 2 months (excluding duration of cell wall thickening). Hence, more than a doubling of increment in the induced compared with regular RG period resulted from striking increase in RG rate. This finding is in accordance with Rathgeber et al. (2011), who reported that the extent of RG in silver fir (*Abies alba*) is more related to the rate of cell production than to its duration. Hence, phloem blockage of C transport to belowground organs provided a continuous supply of carbohydrates to sustain increased rate of cambial cell division and wood formation, processes that are considerable energy sinks (Oribe et al., 2003; Koch, 2004; Muller et al., 2011). Several authors (e.g., Simard et al., 2013; Delpierre et al., 2015; Guillemot et al., 2017) also reported that tree growth is limited by C competition between sinks rather than by C resources. During the induced RG period, which extended from August through September, C allocation was primarily restricted to cambial activity and wood formation, because within the study area shoot and needle growth of *P. sylvestris* ceases in June and July, respectively (Swidrak et al., 2013), and apical meristems remained in dormancy state after girdling. Similarly, internal shifts of C allocation were found to affect the second growth peak in *Pinus pinaster*, a species showing a bimodal RG pattern (Garcia-Forner et al., 2019). It has to be considered that water availability is the primary growth-limiting environmental factor within the study area (Pichler and Oberhuber, 2007; Oberhuber et al., 2011; Gruber et al., 2012) and therefore increase in precipitation by *c.* 30% during the induced compared with regular RG period might also be involved in RG increase at the xeric study site. However, air temperature was found to be inversely related to RG and stem water deficit of *P. sylvestris* within the study area (Oberhuber et al., 1998, 2015; Oberhuber, 2017). Therefore, strikingly higher temperatures during the induced compared with regular RG period (+6.7°C) increased evapotranspiration, which probably compensated the stimulating effect of higher precipitation on RG for the most part. Lack of increase in SWC despite higher precipitation is explained by increase in evapotranspiration together with surface run-off on the steep slope during high precipitation events in summer.

Although maximum daily RG in conifers from cold environments is related to photoperiodic growth constraint to allow xylem differentiation to be completed before early frosts occur (e.g., Rossi et al., 2006; Gruber et al., 2009), this does not apply for *P. sylvestris* on drought-prone sites (Swidrak et al., 2011). Because both RG periods are characterized by (*i*) the same duration (about 2 months), and (*ii*) the same length of time until the maximum daily RG rate was reached (about 2 weeks), an endogenous control of cambial activity and tracheid differentiation as an adaptation to extreme environmental conditions (drought stress, nutrient deficiency) can be assumed.

### Wood Anatomical Changes After Phloem Blockage

Wood formation is a highly C demanding process (Koch, 2004; Simard et al., 2013; Cuny et al., 2015) and low sink priority of the cambium for C was frequently reported (Cannell and Dewar, 1994; Ericsson et al., 1996; Muller et al., 2011; Heinrich et al., 2015). Therefore, we expected a clear role of C accumulation above girdling on wood anatomical traits after interruption of basipetal C transport by girdling. This hypothesis was confirmed by missing significant differences (*p* > 0.05) of wood anatomical parameters (i.e., CLD, CA, CWT, and CLD:CWT) between earlywood and latewood tracheids after girdling, i.e., the characteristic wood anatomical pattern in conifers (thin-walled earlywood cells with large lumina vs. thick-walled latewood cells having narrow lumina) was not sustained as a result of increased C supply. Significant decrease in CLD and CA in earlywood after girdling can be explained by (*i*) decrease in xylem sap flow frequently reported to be a consequence of physically blocking phloem transport (e.g., Zwieniecki et al., 2004; Lopez et al., 2015; Oberhuber et al., 2017) and leading to decrease in turgor pressure and cell enlargement (Cabon et al., 2020), and/or (*ii*) high C availability resulting in faster cell wall deposition, which reduces cell enlargement time (Cartenì et al., 2018). That cell enlargement time primarily explains CLD in conifers was reported by Cuny et al. (2014). Increase in osmotically active C compounds after phloem blockage are required to produce adequate wall-yielding turgor pressure for cell expansion (Pantin et al., 2013; Steppe et al., 2015) and explain significant increase in CLD and CA and concomitant decrease in wood density (increase of CLD:CWT ratio) of latewood tracheids formed after girdling. Furthermore, significant increase in CWT and wood density (decrease in CLD:CWT ratio) in earlywood tracheids after girdling can also be regarded as an outcome of C accumulation above the girdling zone, because C availability is a constraint of cell wall formation (Cuny et al., 2015; Deslauriers et al., 2016; Winkler and Oberhuber, 2017; Cartenì et al., 2018).

Below the girdling zone cell differentiation ceased after interruption of C transport by phloem blockage causing a striking decrease in CWT in last few latewood tracheids (resulting in increase of CLD). Although a decrease in CWT in last latewood tracheids frequently occurs (Arzac et al., 2018; Pacheco et al., 2018; Castagneri et al., 2020), a direct link to decline in C availability by girdling can be deduced from observations that (*i*) cell wall thickening in *P. sylvestris* lasts until September within the study area (Gruber et al., 2010), (*ii*) reduction in C supply due to insect defoliation similarly reduces cell wall thickening in conifer species (e.g., Axelson et al., 2014; Castagneri et al., 2020; Peters et al., 2020), and (*iii*) cell wall thickening has a considerable C requirement (e.g., Cuny et al., 2015).

## Conclusion

Our experimental study revealed that manipulation of stem C status in young *P. sylvestris* trees by phloem blockage triggered bimodal RG above the girdling zone and strongly affected xylem cell differentiation. Results indicate that endogenous control over C allocation, which is most likely hormonally mediated, is a key driver of RG and tracheid differentiation in addition to exogenous factors, i.e., water availability. It is reasonable to assume that a high C sink-strength of belowground root growth and/or storage organs develops early during the growing season as an adaptation to extreme site conditions, i.e., frequent drought stress and low nutrient availability of the substrate, prevailing within the study area. We conclude that tree species showing higher plasticity in RG than *P. sylvestris* will be at an advantage in the long term, because more variable environmental conditions are predicted under future climate change.

## Data Availability Statement

The raw data supporting the conclusions of this article will be made available by the authors, without undue reservation.

## Author Contributions

WO conceived and designed the experiments, and coordinated the research project. AL-W and DS measured and compiled wood anatomical data. All authors were involved in data analyses, and WO wrote the manuscript.

## Conflict of Interest

The authors declare that the research was conducted in the absence of any commercial or financial relationships that could be construed as a potential conflict of interest.
