# Supplementary material for: Triggering Bimodal Radial Stem Growth in Pinus sylvestris at a Drought-Prone Site by Manipulating Stem Carbon Availability
Source: Front Plant Sci. 2021 May 28;12:674438. doi: 10.3389/fpls.2021.674438 (PMC8193578; doi:10.3389/fpls.2021.674438)
Supplement: Supplementary file 1 [file Image_1.pdf]

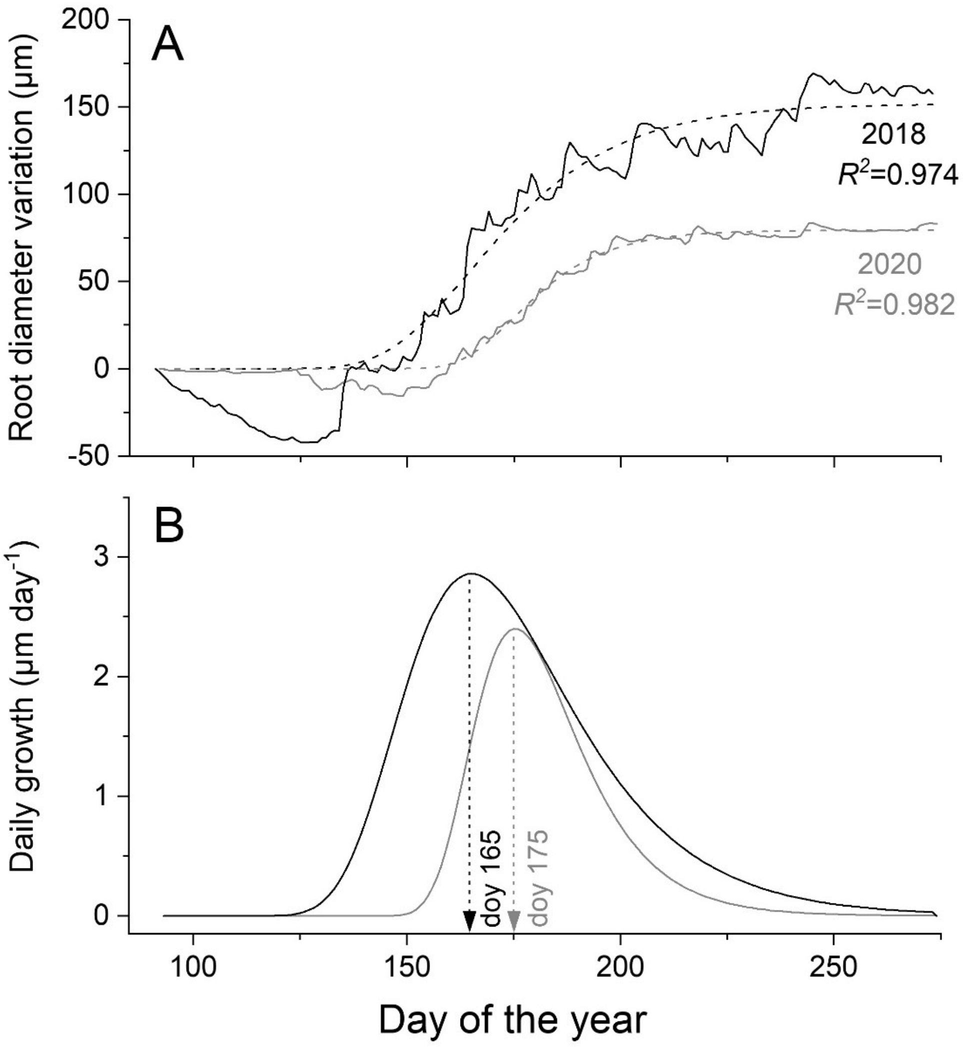

Supplementary Figure 1 | Time series of mean daily dendrometer records of roots (diameter *c*. 10 mm) of mature *Pinus sylvestris* trees. Root growth in 2018 (n=2) and 2020 (n=2) is shown in black and grey lines, respectively. Modelled intra-annual growth by applying the Gompertz function is indicated by dashed lines **(A)**. Daily growth calculated from Gompertz function is depicted in **(B)**. Peaks in daily growth are indicated (doy = day of the year).
